# A Review on the Carbonation and Chloride Penetration Resistance of Structural Lightweight Aggregate Concrete

**DOI:** 10.3390/ma12203456

**Published:** 2019-10-22

**Authors:** José Alexandre Bogas, Sofia Real

**Affiliations:** Civil Engineering Research and Innovation for Sustainability (CERIS), Instituto Superior Técnico, Universidade de Lisboa, 1049-001 Lisboa, Portugal; sofia.real@tecnico.ulisboa.pt

**Keywords:** lightweight aggregate concrete, durability, carbonation, chloride ingress, service life

## Abstract

This paper presents a comprehensive review on structural lightweight aggregate concrete (SLWAC) durability. The main transport properties and degradation mechanisms of reinforced concrete are addressed, namely, carbonation and chloride attack. The influence of the main composition parameters, such as type of aggregate, type of binder and water/binder ratio, as well as the influence of cracking, are also analysed. Finally, the current knowledge of SLWAC’s service life prediction is assessed. Although the knowledge of SLWAC’s durability behaviour is still limited, investigation works performed either in laboratory or in real environments indicate that SLWAC can have similar to better durability performance than normal weight concrete, especially when the same strength level is considered. The importance of the quality of the paste over the characteristics of the lightweight aggregates is highlighted. Durability standardization regarding SLWAC is still insufficient and is one of the main gaps of current knowledge. The objective of this review is to foster a better understanding on the durability and service life prediction of SLWAC, contributing to a greater confidence in using this type of concrete.

## 1. Introduction

Although structural lightweight aggregate concrete (SLWAC) has been used for more than 2000 years, it only assumed greater relevance in construction in the middle of the twentieth century, especially in solutions where the self-weight is a relevant factor, such as in high-rise buildings, shell and slender structures, long-span bridges and rehabilitation works. Since then, SLWAC has been the subject of ongoing technologic and scientific developments, especially in the last three decades, after the technological development of high-performance concrete, the research and application of new admixtures, the advances in manufacturing new lightweight aggregates (LWA) and, most of all, due to the more exigent durability requirements in marine structures, where SLWAC may double the percentage of load reduction [1,2].

However, despite research efforts, knowledge is still limited, especially when compared to that of normal weight concrete (NWC). On the one hand, the higher production costs, the lower quality of the first generation of SLWAC and the less confidence in its application have hindered its affirmation in construction. On the other hand, SLWAC properties can greatly vary depending on the type of LWA and concrete composition, which makes their characterization more complex [3,4,5].

Regarding the durability behavior of SLWAC, knowledge is even less consolidated. Research carried out in this domain shows that the durability and service life performance of SLWAC are influenced by various factors, such as the type of LWA, concrete composition, exposure and testing conditions, water content and deterioration mechanism [6,7], which make its interpretation and general characterization difficult.

Nevertheless, the adequate durability of SLWAC has been proven through the excellent performance evidenced by this material applied in old structures. The vessel production during the world wars, which have been subjected to aggressive environmental conditions for more than a century [1,8,9] evidences the performance of SLWAC in harsh marine environments. The use of SLWAC in the shipbuilding industry was also of great importance in the former Soviet Union. After 15–40 years of navigation experience, no significant corrosion phenomenon was observed [10].

A survey of the Concrete Society [11], which summarizes the use of SLWAC in road structures in North America, the former Soviet Union and Western Europe, indicates that the durability of SLWAC is at least equivalent to NWC.

One of the most significant examples of the durability of SLWAC is attributed to the William Preston Lane Jr. Memorial Bridge, built in 1952, over the Chesapeake Bay in Annapolis, Maryland. The suspension span was built with SLWAC of 24 MPa and water/cement ratio (w/c) of 0.4, while the approach spans were built in NWC [12,13]. In 1975, petrographic and ultrasonic analysis, showing the notable integrity of SLWAC and high deterioration of NWC, led to the option of rebuild the NWC spans with SLWAC.

Mays and Barnes [14] carried out a comprehensive examination of 40 structures in the United Kingdom, including bridges, parking decks and buildings, which indicated that SLWAC was no less durable than NWC. Thienel et al. [15] also reported good durability against freezing and thawing, carbonation and chloride attack of different types of SLWAC from 15 structures in Germany. Both studies indicate that an eventual poor durability of SLWAC may be related to its production process and greater tendency for aggregate segregation.

The durability of SLWAC has also been demonstrated by the Norwegian experience of its application in marine structures, namely, long-span bridges and offshore structures [14,15]. Various investigations on the durability performance of SLWAC in these structures, including laboratory and in-situ tests, suggested that the chloride penetration resistance in SLWAC tends to be at least as high as in NWC [16,17].

However, despite some examples of good performance, knowledge on the durability behavior of SLWAC is still scarce and the mechanisms of deterioration are less dominated than in conventional NWC. Nowadays, due to its more porous nature, SLWAC is still simply seen as less durable than NWC, regardless of the type of aggregate and concrete composition. For this reason, some normative documents conservatively suggest poorly substantiated thicker concrete covers for SLWAC than for NWC, for a given strength level, without accounting for the concrete type and deterioration mechanism [18,19,20]. Moreover, prescribed compositions by the main European standards for a required service life are only defined for NWC or roughly adjusted to SLWAC, without a broad experimental basis [21,22]. As a result, the durability and service life assessment of SLWAC have been the subject of intensive research during the last years, which contributes to a greater confidence in using SLWAC.

This paper discusses most relevant advances in the durability of SLWAC subjected to two of the most frequent and serious concrete deterioration mechanisms; carbonation and chloride induced corrosion. First, the main transport properties of SLWAC, which directly affect its durability are reviewed considering the main differences for NWC. Then, the most relevant findings and up-to-date investigation concerning the carbonation and chloride penetration resistance of SLWAC, as well as its service life performance, are discussed. The objective of this review is to foster a better understanding of the durability and service life prediction of SLWAC and highlight some of the future research needs in this domain.

## 2. Transport Properties

Transport properties, as well as degradation mechanisms, are strongly influenced by the concrete microstructure, which depends on the type, dimension, shape and distribution of their phases [23]. LWA exhibit a high porosity microstructure. The dimension of the interconnected micropores of the aggregate particles (ranging from 1 to 300 µm) is about two to three orders of magnitude higher than the dimension of the pores of the cement paste. Therefore, due to their bigger pore size and open pore structure, LWAs are expected to possess higher penetrability than the cement paste.

Zhang and Gjørv [24] studied the diffusivity of different types of LWA, having concluded that their average diffusivity corresponds to cement pastes with a w/c of 0.9. However, the analyses were carried out on broken particles. As such, on particles with dense surface layers, typical of expanded LWA, lower diffusivity is likely to be obtained.

The idea that the use of porous aggregates leads to higher penetrability of deleterious agents into concrete, resulting in lower durability, has generally been accepted. However, this assumption is highly questionable, as there is evidence suggesting that the permeability of SLWAC is not necessarily higher than that of NWC.

### 2.1. Permeability

**Figure 1:**
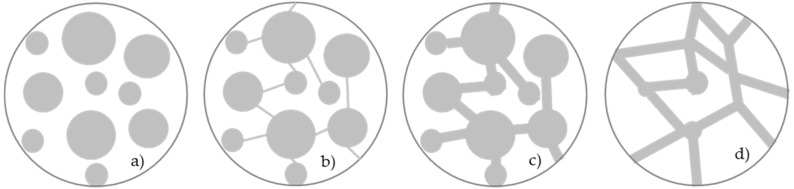
Schematic representation of porosity vs permeability (adapted from [25]): (**a**) high porosity/negligible permeability; (**b**) high porosity/low permeability; (**c**) high porosity/high permeability; (**d**) high porosity/high permeability.

The permeability of concrete is determined by the characteristics of the matrix, the aggregates and the matrix-aggregate transition zone. A permeable material is not necessarily more porous. As schematically shown in Figure 1, pore connectivity is an essential requisite for permeability. In other words, a material can be highly porous and still exhibit low permeability, or have high permeability and display low porosity [1,25].

In SLWAC, the LWA particles are isolated and dispersed throughout the concrete and usually surrounded by cement paste with low pore connectivity. In cement pastes with a medium degree of hydration and a w/c lower than 0.5, a matrix with a closed pore structure can be expected [26]. As such, SLWAC should behave as a system represented in Figure 1a,b [25]. Therefore, although SLWAC is a highly porous material, this does not necessarily mean that exhibits high permeability.

On the other hand, the aggregate-paste interfacial transition zone (ITZ) may also play an important role in the permeability of concrete. In fact, for the usual volume of aggregates in concrete, there is a high probability of intersection and connectivity between ITZ and subsequent establishment of continuous passages for the percolation of substances [27]. On this regard, in SLWAC, the ITZ is usually of better quality than in conventional concrete [28,29,30]. Actually, in SLWAC, the ITZ tends to be, at least, as dense as that of the bulk paste [31]. This is attributed to the penetration of hydrated products in the porous LWA surface (mechanical interlocking) and the higher hydration of these regions due to internal curing [6,29]. Naturally, the greater refinement and the improved quality of the ITZ are more relevant for less dense LWA with a more porous outer layer, in which mechanical interlocking is more effective [32].

Huang, et al. [33] recently analysed the ITZ microstructure of 50-year SLWAC samples collected from the deck of the Nanjing Yangtze River Bridge, in China. SLWAC was produced with w/c of 0.44 and 29 MPa of compressive strength. The authors confirmed the penetration of hydration products in the inland zone of porous LWA, which improved the connection between the cement matrix and the LWA, and hence, the quality of the ITZ. However, in denser regions of the surface of LWA, SLWAC behaved like NWC and, when loaded, cracks propagated through the more porous ITZ. In fact, a water film with higher w/c may develop near the surface of denser LWA (wall-effect) and hydration products of relatively larger crystals are formed. Nevertheless, according to Huang et al. [33], the porous nature of the ITZ was more evident in NWC than in dense SLWAC.

Therefore, despite its high permeability, the use of LWA proves very advantageous, by providing a high-quality ITZ.

The higher elastic compatibility between the aggregate and the paste in SLWAC also leads to low levels of microcracking throughout the matrix. Moreover, SLWAC usually presents lower w/c and lower volume of aggregates than NWC of equal strength, which results in denser cement matrixes and lower percentage of ITZ [34]. In addition, as mentioned, the recognized internal curing provided by LWA also contributes to higher hydration and densification of the inner paste [29,35,36]. These are the main reasons for SLWAC presenting equal or lower permeability than NWC [34,37].

Several authors have underlined the importance of the ITZ for the potential durability of SLWAC [1,13,38]. According to Vaysburd [39], in NWC, the water penetration occurs through the paste and the ITZ, whereas, in SLWAC, the water penetration occurs mainly through the paste, due to the high quality of the ITZ. 

**Figure 2:**
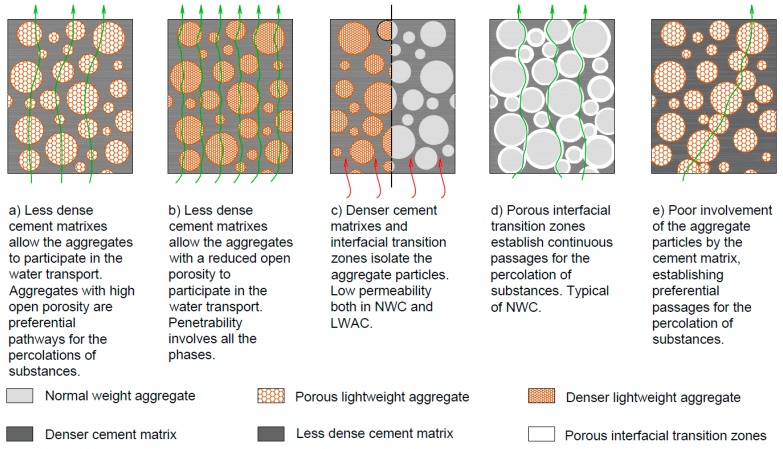
Schematic representation of penetrability of different types of concrete (adapted from [2,25]).

Taking this into account, concrete must be assessed as a whole and not through the sum of the individual contribution of each phase [2,25]. Figure 2 schematizes the penetrability of concrete based on the characteristics of each phase.

In NWC, produced with dense NWA, fluid penetration essentially occurs through the paste or the lower quality ITZ (Figure 2d) [39]. In SLWAC, permeability is affected by the type of aggregate and cement matrix composition. When the matrix is of low quality, the LWA may participate more actively in the permeation process, especially in SLWAC with more porous LWA (Figure 2a,b) [25]. On the other hand, if the cement matrix is dense and the LWAs are properly dispersed and isolated in the cement matrix, their participation in the permeability of SLWAC should be less significant [25,40,41], especially taking into account the high quality of the ITZ in this type of concrete (Figure 2c). As such, the volume of paste in SLWAC should be increased in order to ensure that all LWA particles are surrounded and protected by cement paste. Otherwise, there is a high probability that the LWA particles would not be properly isolated and continuous passages for fluid transport are established (Figure 2e) [27]. The higher amount of fines in SLWAC also counteracts the greater segregation susceptibility of LWA in these concretes [42,43].

According to Bardhan-Roy [44], in SLWAC, the LWAs are dispersed and surrounded by high quality paste, which may result in high resistance to ingress of aggressive substances and fluids. This is attributed to the break in the pore connectivity of the aggregate particles, the more compact cement matrixes for the same strength levels and the lower porosity and microcracking in the ITZ. Similar findings were obtained by other authors [40,41,45,46]. Khorin [45] further attributed the low permeability of SLWAC to the already mentioned higher LWA-paste elastic compatibility and the enhanced bond between the coarse aggregates and the cement matrix.

### 2.2. Water Permeability

In the last decades, both liquid and gas fluids have been used to determine the permeability of SLWAC and NWC. Several authors have demonstrated that the water permeability can be similar in SLWAC and in NWC [2,13,35,37,38,47,48,49,50,51].

Permeability investigations conducted on SLWAC and NWC subjected to the same testing criteria were carried out by Khorin [45], Nishi et al. [46], Keeton [52], Bamforth [53] and Bremner et al. [54]. In spite of the wide range of concrete strength levels, the different intruded substances (gas, water, oil) and the testing techniques applied (specimen size, media pressure and equipment), the permeability of SLWAC was similar or even lower than that of NWC.

Bamforth [53] investigated the permeability of SLWAC produced with fly ash (FA) LWA with a 28-day compressive strength of 49.5 MPa. The tests revealed that both gas permeability and water permeability of SLWAC were slightly lower than those of NWC of similar composition. An experimental investigation carried out by Zhang and Gjørv [38] revealed that the water permeability of high strength SLWAC produced with three types of expanded clay LWA and one type of sintered FA LWA, with w/c of 0.28–0.44 and with strength levels between 57 and 102 MPa, is generally very low and appears to be more dependent on the porosity of the cement matrix than of the LWA.

Hammer [47] performed water absorption and penetrability tests with 4 MPa water pressure on SLWAC produced with FA LWA, with a water/binder ratio (w/b) of 0.3–0.4 and 5–8% silica fume (SF). The results demonstrated that the water penetrability in SLWAC was similar to that of NWC with the same composition. The author concluded that, for low w/c, the porosity of the aggregate did not significantly affect the permeability of SLWAC. This was corroborated by the work of Smeplass and Havdahl [55], which showed that the permeability and long-term water ingress in SLWAC with expanded clay LWA and a w/b of 0.30, was lower than that of NWC of equal composition, even when tested with 4 MPa water pressure. These results corroborate that the influence of the aggregate type on the permeability of SLWAC is insignificant, at least for low w/b. Liu et al. [50] also reported very low water permeability (<10^−13^ m/s) for SLWAC with w/c of 0.38.

In fact, several authors support the idea that the durability of SLWAC, like conventional NWC, is mainly governed by the quality of the cement paste and less by the porosity of the aggregates [38,46,47,56,57,58,59]. This is in accordance with Powers et al. [60], having shown that water permeability is not significant in low w/c pastes.

Nevertheless, the relative importance of the LWA increases when they have a very open porous structure and the omission of a dense outer shell, as occurs in most volcanic LWA [2]. Hossain and Lachemi [61] determined the water permeability of concrete with volcanic pumice LWA and a w/c of 0.45, concluding that the substitution of NWA with LWA led to higher water permeability. Even for the same strength levels, the permeability of SLWAC was 136% higher than in its NWC counterpart. The same findings were obtained by Teo et al. [62], in SLWAC produced with oil palm shell LWA and w/c of 0.33–0.41.

Based on various investigations carried out in Romania, Ionescu and Ispas [48] reported that, for high strength levels, the permeability of SLWAC was similar to that of NWC and, for low strength levels, the permeability of SLWAC was higher than that of NWC. Bologna and Levi [59] emphasize the hypothesis that SLWAC with higher w/c presents higher water penetrability.

Bogas [2] studied the water permeability of SLWAC produced with LWA of distinct porosities (density of 1070 and 1300 kg/m^3^), for w/c of 0.35, 0.45 and 0.65. The water pressure was only 0.5 MPa. In general, the water permeability was similar in SLWAC and NWC with w/c up to 0.45. However, slightly higher water penetration was observed in SLWAC with more porous LWA, which was attributed to the broken LWA particles on the surface of cut concrete specimens. In fact, based on the observation of the inner region of concrete specimens, the water front was not higher near the LWA particles. For concrete with w/c of 0.35, the water penetration was very low, regardless of the type of aggregate. The author concluded that these tests are not adequate to analyse the relative behaviour of different types of concrete with low w/c. For concretes with pastes of lower quality (w/c of 0.65), a higher permeability in SLWAC with more porous LWA than in NWC was confirmed. In this case, the water penetration front was higher under the LWA particles, showing their effective participation in permeability. However, even for this high w/c, the permeability of SLWAC with denser LWA was similar to that of NWC. These findings confirm that highly porous cement pastes allow a greater participation of the aggregates in the water transport.

In the aforementioned study developed by Zhang and Gjørv [38], the authors noticed that a too high cement content possesses an adverse influence on the permeability, resulting in a higher permeability of SLWAC than of NWC of equal strength. This phenomenon was attributed to the significant increase in the stiffness of the cement paste, increasing the elastic compatibility between the aggregates and the paste in NWC and reducing in SLWAC. Following these findings, Chia and Zhang [49,63] analysed the 4 MPa water penetration in both SLWAC and NWC of equal composition. Concrete with a w/c of 0.55 and 28-day compressive strength of 30–40 MPa and high-strength concrete with a w/c of 0.35, 0–10% of SF and 28-day compressive strength of 50–90 MPa were produced. At the strength level of 30–40 MPa, the water permeability of SLWAC was lower than that of the corresponding NWC, due to the better quality of the ITZ and the higher elastic compatibility between the aggregates and the paste in SLWAC. However, the water permeability of the high-strength SLWAC and NWC was similar, which was justified by the increase of the elastic compatibility in NWC. The authors also concluded that, for equal strength level, the permeability of SLWAC was lower than that of NWC. This was explained by the better paste quality on SLWAC of equal strength levels.

However, Al-Khaiat and Haque [64] found similar higher water permeability in SLWAC than in NWC of equal strength. Moreover, Nyame [65] reported that mortars with LWA presented water permeability twice as high as those of mortars with NWA with the same w/c. Both these studies highlight the fact that the participation of the aggregates in the water transport depends on the relative importance of the different phases in the mixture, as depicted in Figure 2.

Actually, due to the extremely low water permeability of common concretes, this test is not very informative of the relative permeability behaviour of SLWAC and NWC. Abbas et al. [66] and Lee et al. [67] highlight that precise and repeatable results in water permeability tests can hardly be obtained in dense common cement pastes. Therefore, Abbas et al. [66] considered oxygen permeability to be a more adequate type of test, requiring less time and resulting in more repeatable results.

### 2.3. Gas Permeability

Concerning gas permeability, some contradictory results have been reported in the literature. On the one hand, after performing nitrogen and oxygen permeability tests in the United Kingdom, Mays and Barnes [14] reported up to three orders of magnitude lower permeability in SLWAC than in equal strength NWC. Bamforth [53], Sugiyama et al. [68] and Ben-Othman and Buenfeld [69] also reported lower gas permeability in SLWAC than in NWC of the same composition.

On the other hand, Bogas [2], Güneyisi et al. [70], Real and Bogas [34], Lydon [71] and Lydon and Mahawish [72] report similar to higher gas permeability in SLWAC than in NWC of the same composition. Gesoǧlu et al. [73] reported 50% higher oxygen permeability in SLWAC with FA LWA and a w/c of 0.3 than in NWC of the same composition. According to CRIC [74], based on a wide experimental program, SLWAC produced with expanded clay and shale LWA, with a compressive strength of 40 MPa and a w/c of 0.6, revealed an oxygen permeability about 1.2–1.5 times higher than that of NWC of the same composition, despite presenting similar water permeability.

The water content plays a major role in gas permeability and diffusivity in concrete. According to FIP [37], the water content in SLWAC is generally higher than in NWC, at least at early ages. This is in line with the findings of Real and Bogas [34]. For the same preconditioning, the authors observed that SLWAC presented slightly higher relative humidity (RH) than NWC of the same composition. According to Smeplass [75], the combination of a higher initial water content and a lower permeability of the matrix in SLWAC with a low w/c may significantly increase its drying period. The drying periods and the time to reach a hygral equilibrium between the two phases tend to be longer [37,76]. This phenomenon is especially relevant in SLWAC produced with fine and coarse LWA [44]. During the drying period, the finer capillary pores in the cement paste force the migration of the moisture from the coarse pores of the LWA [25,75,77]. However, from the moment this water is eliminated, which may occur for an equilibrium RH lower than about 97%, the desorption isotherm curves become identical [25,75]. These particularities of the behaviour of SLWAC during the drying period may have repercussions in gas penetrability.

According to Bogas [2], in low w/c concretes, the decrease of the water content of inner regions is the result of self-desiccation. Given that, in SLWAC, the self-desiccation mechanism is normally offset by the water supplied by the LWA particles, which work as small water containers scattered within the cement paste, the saturation level of the pores remains high, contrary to what commonly happens in NWC. Consequently, the gas permeability of SLWAC may be reduced. Contrarily, in high w/c concretes, the self-desiccation mechanism is of minor relevance and the drying is more effective. In this case, the pore structure of the paste, with a lower degree of saturation, is more permissive to gas ingress and may promote its transport through the matrix to the aggregate particles, which also possess low water content. Depending on the porosity of the internal structure and of the outer shell of the particles, the contribution of LWA to gas permeability should be variable.

According to Lydon [71], the high quality paste enclosing the aggregate particles is of primary importance for low permeability, regardless the high porosity of the LWA. Alternatively, if the porosity of the LWA is relevant, the contribution of the aggregates to the gas permeability is only effective when a sufficient amount of water in the pores of the paste is eliminated, allowing the gas passage. As such, the w/c, the curing process and the type of aggregate are relevant parameters to the gas permeability of SLWAC.

Ben-Othman and Buenfeld [69] performed oxygen permeability tests on concretes subjected to various preconditioning methods, having obtained similar to lower oxygen permeability in SLWAC than in NWC of the same composition. Vieira [78] found that SLWAC with LWA of high porosity and a w/c of 0.3 presented lower oxygen permeability than SLWAC with LWA of high porosity of the same composition. The author also found that SLWAC exhibited lower oxygen permeability than NWC with the same w/c, although the differences were not significant. However, for a w/c of 0.45, SLWAC with LWA of high porosity exhibited a significantly higher permeability, suggesting that these results are related with the water content of the concrete and with the higher participation of the LWA associated to more porous cement pastes.

Real and Bogas [34] performed oxygen permeability tests on SLWAC with four types of LWA with very distinct porosities, different types of binders and w/b of 0.35–0.55. The oxygen permeability coefficient varied between 0.59 × 10^−18^ m^2^ and 30.4 × 10^−18^ m^2^, covering a wide range of low to high permeable concrete. The authors found that, for a w/b of 0.35, oxygen permeability was very low, regardless of the type of aggregate. The influence of the type of aggregate tended to increase with the w/b, due to the increment of porosity of the paste and consequent availability of the LWA to participate in gas permeability. For a w/b of 0.55, the oxygen permeability coefficient of SLWAC with porous LWA was up to 4.5 times higher than that of NWC with the same preconditioning. The participation increased in SLWAC with LWA of lower density (expanded clay LWA) or absence of dense outer shell (FA LWA). Nevertheless, the oxygen permeability of SLWAC with dense LWA was similar to that of NWC of the same composition and preconditioning procedure. However, for oven dry conditions, the authors found that the oxygen permeability of NWC was significantly higher than that of SLWAC, possibly due to microcraking developed in NWC during the drying process. The oxygen permeability coefficient decreased as the applied pressure increased, confirming the well-known Klinkenberg effect, in both NWC and SLWAC. Finally, a poor correlation between the oxygen permeability and the compressive strength was also found, because these properties are differently affected by LWA.

Lydon [71] obtained contrary results when testing the nitrogen gas permeability of SLWAC with sintered FA LWA and NWC with w/c of 0.40 and 0.50, which were water-cured and then dried. In general, all concretes exhibited low permeability, but higher weight loss and permeability were obtained in SLWAC. However, SLWAC and NWC of equal strength presented similar permeability. On another investigation carried out by Lydon and Mahawish [72], the gas permeability of SLWAC with a w/c of 0.5 (subjected to prolonged water curing, followed by air and oven-drying) was slightly higher than that of NWC of the same composition and with the same preconditioning procedure. In these studies, the difference in gas permeability between SLWAC and NWC increased with the severity of drying. These results confirm the role of drying in increasing the accessibility of the LWA particles to the pore structure, despite the high quality of the paste, contributing for higher permeability of SLWAC.

Therefore, the gas permeability of SLWAC seems to be not only influenced by the physical characteristics of the paste and the ITZ, but also by the preconditioning methods. Other important factors, such as the quality of the specimens’ production, the curing conditions and the type of aggregate are likely to affect the results, which may justify the apparent discrepancies found in the literature [2].

### 2.4. Capillary Absorption

Like gas permeability, the capillary absorption of SLWAC is significantly affected by the water content. Hammer and Hansen [79] reported that, if the concrete is not subjected to effective drying before testing, the ingress of water is restricted to a few millimetres from the surface. According to the authors, the water initially retained in the LWA (internal curing effect) compensates for the self-desiccation mechanism and prevents the development of capillary forces, which promotes the penetration of water into concrete. Moreover, due to internal curing, the further hydration may contribute to the reduction of the capillary porosity of SLWAC, reducing the water absorption [29,35,50,80,81].

For concretes with low w/c, Vieira [78] found lower capillary absorptions in SLWAC with highly porous LWA than in NWC, having attributed these results to the better quality of the ITZ and to the fact that the water contained in the aggregate particles decreases the absorption capacity of the matrix. For concretes with lower strength levels, in which the drying and the participation of the LWA particles were more effective, the author reported lower absorptions in NWC than in SLWAC.

However, Punkki and Gjorv [82] documented lower capillary absorptions in SLWAC compared to those in NWC, even when subjected to previous drying. The authors also verified that the behaviour of SLWAC is affected by the initial water content. This study reported lower absorptions in SLWAC with initially dry LWA than with pre-wetted LWA, due to the better quality of the ITZ in the first ones. In fact, in NWC, the ITZ has a decisive contribution to capillary absorption, whereas, in SLWAC, due to the high quality of these regions, the water penetration occurs homogeneously through the paste [39].

**Figure 3:**
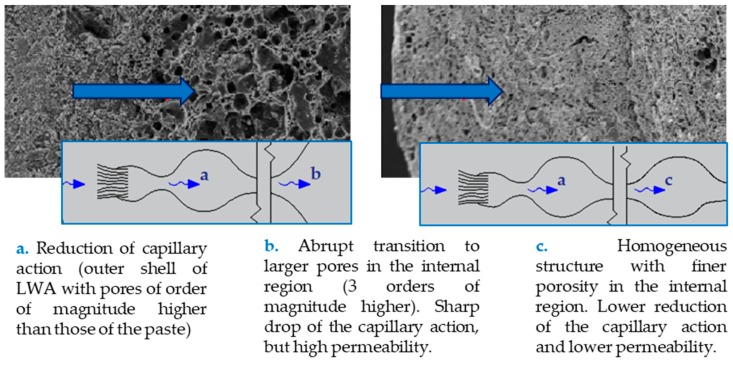
Schematic representation of the transition of porosity between aggregate and matrix (adapted from [29]).

As mentioned, concrete transport properties are directly related to porosity. However, according to Bogas et al. [29], in SLWAC, capillary absorption and permeability are influenced by the porosity of the LWA in different ways. For instance, an abrupt transition from the porosity of the paste to that of the LWA (2–3 orders of magnitude higher) leads to an accentuated drop in capillary action, but increased permeability (Figure 3). In fact, the capillary effect is reduced in pores of high dimensions, as is the case of LWA [35]. According to Smeplass [75], in SLWAC with low w/b, the fine pore system of the cement paste limits the ability of the LWA to contribute to the capillary water transport. However, in SLWAC with high w/b, the pore system is coarser and the capillary resistance of the paste is reduced, allowing the LWA to better participate in water transport. Hammer and Hansen [79] further reported that, as the pore sizes of LWAs are often much higher than those of the paste, the capillary forces in the LWAs are not strong enough to increase the water flow in the paste.

In fact, various authors report similar capillary absorptions in both SLWAC and NWC, inferring that, for low w/c, the participation of the LWA in this transport mechanism is not significant [6,29,35,83]. Real et al. [6] studied the capillary absorption of SLWAC with four types of LWA, different types and volumes of binder and w/b between 0.35 and 0.55. The authors confirmed that, except for LWA with high porosity and superficial cracking or without a dense outer shell, the absorption coefficient was not significantly influenced by the type of aggregate, depending essentially on the quality of the matrix and ITZ. The same was found by Bogas et al. [29], even taking into account very different drying degrees and w/c. The authors concluded that the high porosity of LWA appeared to be of secondary importance to this property. The low influence of LWA was also confirmed by the similar absorption coefficients found in SLWAC produced with distinct volumes of the same type of LWA or with partial replacement of NWA by LWA [29].

However, some authors found that SLWAC showed higher capillary absorptions than NWC [35,50,70,73,84,85]. Mo et al. [85] reported that the capillary absorption of SLWAC produced with oil palm shell LWA and a w/b of 0.33 was higher than those reported by other authors for NWC. The authors assumed that the higher porosity of the LWA led to higher capillary absorption. Gesoǧlu et al. [73] also confirmed the increment of capillary absorption with the increase of the replacement percentage of NWA with sintered FA LWA in concrete with a w/b of 0.32, having also attributed the results to the high porosity of the LWA. 

In spite of their similar behaviour, Real [86] found that the absorption coefficients tended to be slightly higher in lower density LWA. Bogas et al. [29] also confirmed higher capillary absorption in SLWAC with very porous LWA or with fine and coarse LWA than in NWC of equal composition. This phenomenon occurred even for mixtures with high-quality pastes (w/c of 0.35), having been attributed to the direct water uptake of LWA particles near the cut surface of concrete specimens. This was also observed by Liu et al. [35]. Actually, according to Bogas et al. [29], the absorption process in SLWAC in capillary tests develops in three stages. Initially, an accelerated filling of the superficial LWA and voids occurs, due to their direct exposure to water, through which water ingress is easier. This was demonstrated by the higher (in the first 10 min) water absorption found in SLWAC, regardless of the type of LWA. In fact, due to the greater participation of porous LWA near the surface, the source of water penetration can involve the whole section of the concrete sample. Then, the absorption process continues through the accessible pores of the matrix. During this stage, the absorption rate is similar in SLWAC and in NWC. Finally, after the filling of these pores, the absorption is slower and the LWA, which has a coarser porosity than the paste, starts participating in this process. By testing both sectioned and whole specimens, Real [86] confirmed that the first stage found in sectioned specimens can be eliminated in whole specimens.

Naturally, long-term absorption tends to be higher in NWC than in SLWAC, regardless of the type of LWA, because of the greater total porosity of SLWAC [29].

Real et al. [6] also investigated the influence of the type of binder on the capillary absorption of SLWAC. The authors found that the incorporation of a lime filler (LF) or FA resulted in similar performances in both SLWAC and NWC. The replacement of cement with 9% SF led to a reduction of capillary absorption of about 30% in NWC and 15% in SLWAC. The lower effectiveness of SF in SLWAC was attributed to the low contribution of this mineral admixture to the improvement of the already high quality of the ITZ. Furthermore, the authors suggest that LWA could have absorbed part of the SF during mixing, though the LWA had been pre-saturated.

The different findings documented in the literature reveal that there is still a lack of knowledge on this transport mechanism in SLWAC, associated with highly variable behaviours depending on the characteristics of the concrete and the testing methods.

## 3. Carbonation

### 3.1. Influence of the Type of Aggregate

**Figure 4:**
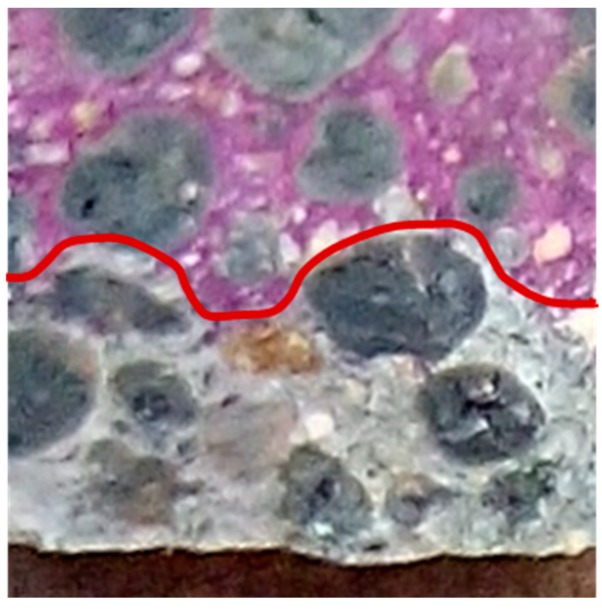
Deeper carbonation front in porous LWA than in matrix [86].

**Figure 5:**
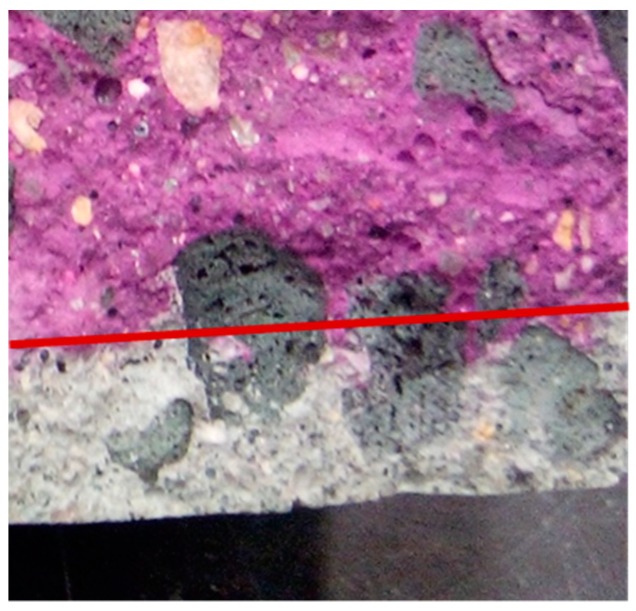
Similar carbonation front in dense LWA and in matrix [86].

The carbon dioxide diffusion rate through concrete is mainly governed by the characteristics of its porous structure. Due to their higher porosity, LWAs present lower carbonation resistance than NWA [9,25,40], which favours the carbon dioxide (CO_2_) diffusion. Moreover, contrary to the cementitious matrix, LWAs do not have CO_2_ binding capacity [2,86]. In fact, Schulze and Günzler [57] confirmed a deeper carbonation front in the region of LWA than through the surrounding bulk cement matrix. This was also observed by Real [86], when SLWAC was produced with porous LWA (Figure 4). However, when less porous LWA with a dense outer shell was used, the carbonation front was not necessarily higher through the aggregate (Figure 5).

**Figure 6:**
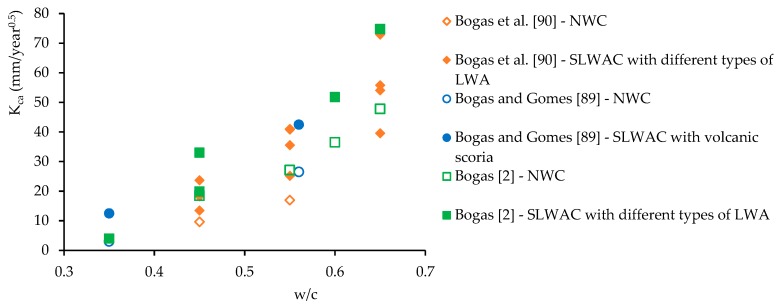
Carbonation coefficient vs w/c, according to various authors [2,89,90].

In general, a higher carbonation is observed in SLWAC than in NWC [2,78,87,88,89,90] (Figure 6). Brown and Beeby [87] observed mean carbonation depths about 90% greater in SLWAC with FA LWA than in NWC of equal w/c. Gündüz and Uğur [88] mentioned the decrease of the carbonation rate with the reduction of the LWA/cement ratio in SLWAC with pumice LWA, which demonstrates the effective participation of LWA. Zhao et al. [91] also found that by increasing the volume ratio of fine aggregate to total aggregate in SLWAC with coarse and fine waste clay brick aggregates the carbonation rate after 28 days was accelerated. The results were also attributed to the increased pozzolanic activity of these aggregates, due to the increment of their specific surface area, which reduced the amount of calcium hydroxide available for carbonation.

Bogas and Gomes [89] studied the carbonation behaviour of SLWAC with coarse and fine volcanic scoria LWA and w/c of 0.35 and 0.65. The authors found that the carbonation rate in SLWAC was greater than in NWC of equal composition, independent of the w/c, due to the high open porosity or lack of dense aggregate outer shell. Vieira [78] also obtained higher carbonation depths in SLWAC with expanded clay LWA of high porosity than in NWC of equal w/c. Nonetheless, NWC and SLWAC with denser LWA presented similar carbonation depths.

According to Bogas et al. [90], the participation degree of LWA in carbonation does not only depend on the characteristics of the LWA, but also on the quality of the surrounding paste. LWA should have a greater influence on carbonation when encased in a high w/c paste than in a denser matrix. Many authors have highlighted the importance of an effective dispersion and involvement of the LWA particles by a high quality paste, in order to avoid the establishment of diffusion bridges between the concrete surface and the steel reinforcement through the LWA particles, increasing the risk of corrosion [37,40,57,58,92]. Taking this into account, Vaysburd [39] suggests that, for LWA with lower density than 1300 kg/m^3^, the thickness of the concrete cover needs to be at least two times higher than the maximum LWA size. However, this recommendation may be too conservative and should also contemplate the quality of the paste. In fact, some authors have reported that high-carbonation resistance SLWAC can be achieved through low w/c pastes [57,93]. Bogas [2] assessed the carbonation resistance of SLWAC with various types of LWA, types and amounts of binder and w/b of 0.35–0.65. The author concluded that, for w/c lower than 0.4, the carbonation depths in SLWAC and NWC were insignificant, and these concretes exhibited similar carbonation performance. However, for higher w/c, the carbonation depth was significant and the carbonation resistance of NWC was higher than that of SLWAC.

Various authors have reported lower carbonation in SLWAC than in NWC of the same strength [94,95,96,97]. In fact, for the same strength level, SLWAC generally has a denser matrix than NWC, due to the higher cement content and lower w/c, which consequently decreases permeability. Thus, both the amount of carbonatable substances and the protective capacity of the matrix tend to be higher in SLWAC than in NWC of the same strength level, contributing to lower carbonation depths [13]. Lo et al. [94] confirmed the higher carbonation resistance in SLWAC with expanded clay LWA than in NWC of equal strength. However, SLWAC carbonation was slightly lower in NWC and the participation of LWA increased in pastes with open pore structures. Lo et al. [95] attributed the higher carbonation resistance of SLWAC with FA LWA than that of NWC of the same strength to the better quality of the ITZ. Bremner et al. [97] measured the carbonation performance of SLWAC and NWC at various strength levels. The authors concluded that the carbonation depths were lower in SLWAC than in NWC of equal strength, having mentioned that the high quality paste was a fundamental aspect for an adequate carbonation behaviour. Compared to equal compressive strength NWC, Al-Khaiat and Haque [76] observed marginally higher carbonation depths in SLWAC with coarse and fine FA LWA. However, for curing periods greater than 1 week, associated to pastes with a higher hydration level, the carbonation resistance of both SLWAC and NWC was comparable. Considering an equal strength level, Haque et al. [96] referred similar carbonation depths in SLWAC with coarse FA LWA and in NWC.

The influence of water content on CO_2_ diffusion in SLWAC is manifested in two ways. On one hand, as mentioned (Section 2.3), the drying period is longer in SLWAC than in NWC. Therefore, a higher carbonation resistance is expected, at least up to the period until the hygral equilibrium is attained between the environment and concrete. On the other hand, as soon as the RH of concrete is reduced, the water stored in LWA migrates to the paste, allowing them to participate in gas diffusion [2,98]. After drying, the resaturation of LWA is difficult.

Contrary to NWC, a poor correlation between the compressive strength and carbonation resistance is reported, as both properties are differently affected by LWA [90]. For example, for SLWAC with low w/b, the type of aggregate has little influence on the carbonation resistance, whereas it significantly influences the compressive strength [90]. This must be taken into account when the durability of SLWAC is specified based on compressive strength.

The previously discussed aspects highlight the more complex carbonation behaviour of SLWAC than that of traditional NWC. The great diversity of commercially available LWA allows the production of SLWAC with a wide range of density and compressive strength classes, which makes the general characterization of the SLWAC carbonation behaviour difficult. In this context, Bogas et al. [90] developed a comprehensive work involving a wide range of concrete compositions, which allowed a better understanding and characterization of the carbonation behaviour of common SLWAC, as discussed in the next sub-chapters.

### 3.2. Biphasic Carbonation Model

**Figure 7:**
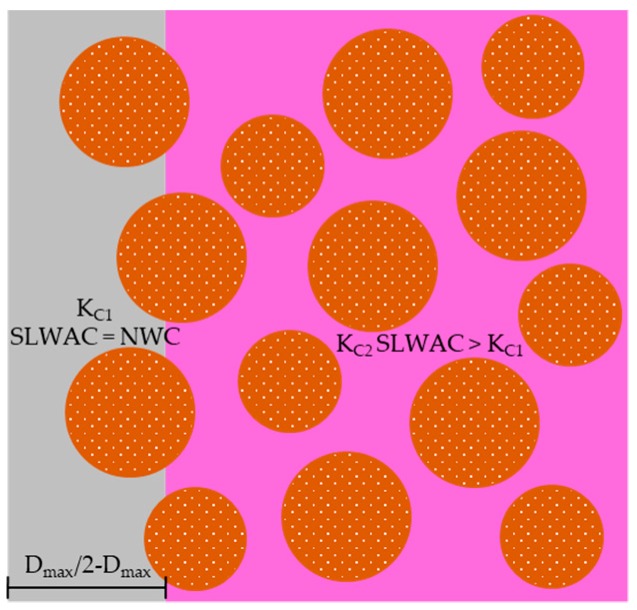
Biphasic carbonation model for SLWAC suggested by Bogas et al. [90]. The carbonation coefficient during the first stage is similar in SLWAC and NWC, while in the second stage tends to increase as the LWA porosity increases.

Based on an extensive experimental work involving SLWAC with four types of LWA of distinct porosities, nine types of binders and three w/b, Bogas et al. [90] suggested a biphasic model to better describe the carbonation behaviour of SLWAC (Figure 7). According to this model, in the first stage, until the carbonation front overcomes a *Dmax*/2–*Dmax* thick layer (maximum diameter of coarse aggregate, Figure 7), where the presence of the coarse LWA is limited, due to the well-known wall-effect, the carbonation rate of SLWAC is similar to that of NWC of the same composition, depending essentially on the properties of the paste. In the second stage, the participation of the LWA becomes effective and the carbonation rate of SLWAC varies according to the density of LWA. This model is not valid when the carbonation occurs through the casting surface, which is rarely the case in real concrete structures. Based on this model, the authors typified the carbonation behaviour of SLWAC in both stages as a function of the w/c and LWA density class. According to the authors, this model can explain some apparently contradictory results presented in the literature, namely, when comparing accelerated carbonation results with natural exposure tests with small carbonation depths. In fact, as the carbonation mechanism is a slow process for SLWAC with pastes of good quality; the transition point is only long-term attained and the performance of SLWAC may be identical to that of NWC for a reasonable service life.

### 3.3. Influence of the Type of Binder

Bogas et al. [90] found that the partial replacement of original Portland cement (OPC) with different types and volumes of mineral admixtures, namely SF, FA and LF (6–9% SF, 15–30% FA, 15–30% LF and 30–50% SF + FA), increased the carbonation rate of SLWAC and NWC. Moreover, no significant improvements were found when these admixtures were considered as addition materials, for the same cement content. In fact, the authors concluded that the potential contribution of these admixtures for the carbonation rate could be disregarded. Taking this into account, general equations were suggested to determine the carbonation coefficient as a function of the w/c and type and class of LWA, regardless of the type and amount of binder (Table 1, [90]). These expressions are defined for the different stages of the biphasic model proposed by the authors. Lo et al. [94] investigated the accelerated carbonation of SLWAC with expanded clay LWA, 25% FA and 5–10% SF. The authors also concluded that the carbonation rate in SLWAC with 25% FA was comparable to that of SLWAC with OPC. However, the authors found higher carbonation rates in ternary mixtures with FA and SF, which means that, in this case, the refinement of the paste microstructure was less relevant than the reduction of carbonatable substances. Gao et al. [99] studied the influence of 20–30% OPC replacement of coarse FA and pulverized FA on the accelerated carbonation resistance (20% CO_2_) of SLWAC with w/b of 0.31, up to 56 days. The carbonation depth and pH solution in SLWAC with FA was higher than in reference SLWAC with OPC. However, the carbonation depth in SLWAC with 20% PFA was only 78% of that of reference SLWAC. Concrete with 30% PFA still showed lower carbonation depth than reference SLWAC, but higher than with 20% PFA. The authors attributed the results to the fact that pulverized FA and granulated blast furnace slag were finer than cement, leading to less porous cement matrixes. In another study, Huberorá and Hela [100] analysed the durability of SLWAC with expanded clay LWA, w/b of 0.33–0.35 and strength class LC30/33-LC35/38, exposed for 12 months to carbonation environment. The mixtures incorporating 40% FA were more resistant than those with also 5% SF or with 40% LF, by volume of cement.

### 3.4. Carbonation Resistance in Real Environment

Over the last decades, research concerning carbonation in real structures [1,14,15,102,103] and in specimens subjected to long-term real environmental conditions [86,104,105] has been conducted. Some relevant information on these investigations is summarized in Table 2.

Holm et al. [102] measured the carbonation depth on cores drilled from concrete ships built during World War II. The cores were extracted from a zone ranging the waterline to as much as 5 m above the maximum water level. Despite being exposed to an aggressive marine environment for more than five decades in the Chesapeake Bay at Cape Charles, Virginia, on average, the carbonation depth was 1 mm, 1–2 mm and non-existent, in specimens taken from the main deck, the wing walls and the hull and bulkheads, respectively. These results were attributed to the high cement content and high-quality of the concrete.

Holm et al. [103] and Holm and Bremner [1] investigated the long term carbonation of concrete in the Chesapeake Bay Bridge, after 35 years exposure. The carbonation depths reached 2–8 mm and 2–13 mm, in cores extracted from the top and from the underside of the bridge deck, respectively. The authors explained that the higher carbonation depth on the underside was due to the increased gas diffusion associated with the drier concrete on this side of the bridge, as well as the reduced carbonation depth on top of the bridge deck, which was covered with 36 mm asphalt wearing course that should have inhibited drying. Furthermore, the authors reported the results of 15-year-old exposed deck surface of the Interchange Bridge at Coxsackie, which revealed only 5 and 10 mm carbonation depths in cores from the top surface and from the bottom, respectively. 

Taking into account the analysis of 19-year-old Japanese bridges and viaducts, Ohuchi et al. [106] measured average 13 and 16 mm carbonation depths in SLWAC specimens with compressive strengths of 23 and 26 MPa, respectively. Bandyopadhyay and Swamy [104] analysed the durability of SLWAC with expanded shale LWA, exposed to an industrial environment for 2 years. No evidence of corrosion was found in SLWAC with w/c between 0.45 and 0.82, even though the specimens only possessed a 13 mm concrete cover. In high strength SLWAC with w/c lower than 0.53, the carbonation depth was under 1 mm. Noteworthy is that the carbonation depth measured in these long-term studies were within the stage 1 suggested in the biphasic model of Bogas et al. [90] (Section 3.2). This may justify the excellent carbonation performance of SLWAC under real exposure reported in literature.

Thienel et al. [15] investigated 17 different types of SLWAC obtained from 15 structures in service, in Germany, after 10 to 30 years of exposure to natural environment. The authors observed carbonation coefficients lower than 5 mm/year^0.5^.

Salem et al. [105] observed lower carbonation in SLWAC with expanded clay LWA and a w/c of 0.5 than in NWC with the same w/c, after being subjected to natural environment for 16 and 32 months. The authors attributed the results to a possible modification of the porous structure of SLWAC, due to pozzolanic reactions between cement and LWA, and also to the denser and thinner ITZ.

Taking into account six concrete structures in the United Kingdom, Mays and Barnes [14] reported 6–25 mm carbonation depths in SLWAC subjected to natural environment for 20 years. Considering the same strength level, the carbonation depth of SLWAC was hardly higher than that of NWC.

Mircea et al. [107] assessed the durability and service life of 260 beams, with compressive strengths of 30–50 MPa, having been exposed to laboratory, urban, marine and industrial environments, for a period of 10–12 years. The authors reported that the carbonation depths in SLWAC with expanded clay were comparable to those of NWC, for cement contents higher than 400 kg/m^3^. However, for lower cement contents, SLWAC beams exhibited carbonation depths 10 to 30% higher than those with reference NWC. In another study, Short and Kinniburgh [58] found that NWC and SLWAC of high w/c, exposed to natural environment for 3 years, presented average carbonation depths of 1.6 and 2.5 mm, respectively. These two research works demonstrate that the participation of LWA in the carbonation mechanism is more effective in pastes with an open pore structure. Swamy and Jiang [93] observed that the carbonation depths increased significantly with the volume replacement of natural sand with expanded slate fines, after 10 years of storage in external environment. In this case, the stage 1 of the biphasic model of Bogas et al. [90] is disregarded.

Bogas [2] subjected SLWAC with various types of LWA and w/b of 0.35 and 0.45 to XC4 environment, in the Setubal port facilities, Portugal. After 2 years of exposure, the carbonation depth was only up to 2 mm, within the stage 1.

Real [86] performed an extensive experimental campaign, involving the exposure of hundreds of SLWAC specimens with different types of LWA and binder and w/b of 0.55 and 0.65, in four different environmental conditions. After 3 years of exposure, the author observed that SLWAC with denser LWA and NWC of the same composition presented similar carbonation depths, independently of the environmental exposure conditions. However, due to the high w/b, SLWAC with more porous LWA presented higher carbonation depths. As shown in Table 2, the carbonation front of lean mixtures was higher than the transition point corresponding to stage 1 of the model presented by Bogas et al. [90]. The author found that both natural and accelerated carbonation tests presented the same tendencies concerning the influence of the main composition parameters [86,90].

Though there have been some breakthroughs in the understanding of the carbonation resistance of SLWAC, namely the biphasic carbonation model suggested by Bogas et al. [90], the behaviour of SLWAC has yet to be completely grasped, requiring further research.

## 4. Chloride Ingress

### 4.1. Influence of the Type of Aggregate

In the last decades, various investigations on chloride ingress have been performed under laboratory conditions [6,7,49,73,84,89,108,109,110].

**Figure 8:**
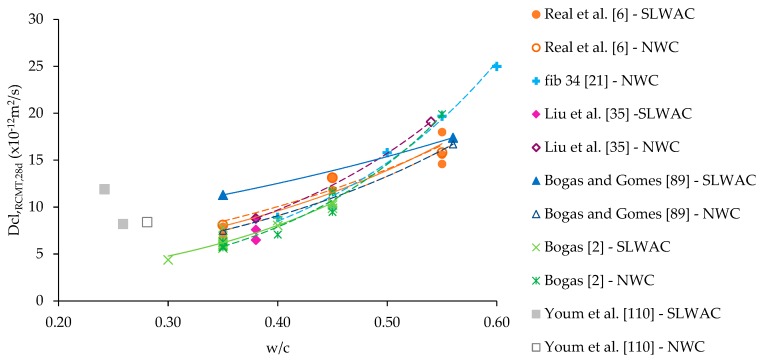
Chloride migration coefficient as a function of the w/c, according to various authors [2,6,21,35,89,110].

A higher chloride diffusion in SLWAC would be expected, because, despite its higher porosity, LWA does not have chloride binding capacity [89]. However, various authors reported that the chloride penetration resistance of SLWAC tends to be at least as high as that of NWC [6,35,49,50,51,70,108,110,111]. This is demonstrated in Figure 8, in which the chloride migration coefficient from rapid chloride migration tests (RCMT), performed according to NT Build 492 [112], were similar in SLWAC and NWC, for different w/c. In fact, though LWA tend to be more permeable than the surrounding matrix, some studies suggest that the protection provided by the paste, namely of high compactness [25,40,41], and the better quality of the aggregate-paste ITZ in SLWAC [1,13,38,79] can lead to similar chloride penetration resistance in both SLWAC and NWC of the same composition [9,25]. Thus, chloride penetration should be essentially governed by the properties of the matrix [25,35,51].

Kayali and Zhu [108] immersed SLWAC slabs with sintered FA LWA and a w/b of 0.24 in a 2% chloride solution, for over 15 months. The authors found that SLWAC slabs exhibited the least amount of free chloride ion concentration at a 5 mm depth, independently of the immersion days. The authors suggest that the LWA might have acted as protective reservoirs, absorbing the chloride solution, and as such, preventing the chloride ions from reaching the steel surface.

Real et al. [6] confirmed similar RCMT chloride migration coefficients in SLWAC and in NWC of equal composition despite the consideration of concretes with very different types of LWA, distinct types and amounts of mineral admixtures and w/b of 0.35–0.55. Similar conclusions were obtained by Liu et al. [35] in SLWAC with expanded clay LWA and a w/c of 0.38, and by Liu et al. [111] in SLWAC with expanded clay LWA and w/b of 0.3 and 0.45. Youm et al. [110] also reported a similar trend, taking into account RCMT in SLWAC with expanded slate and a w/b of 0.25. In addition, Real et al. [6] also found that the chloride migration coefficient was little affected when concrete was tested with different volumes of coarse LWA, which confirms that the chloride migration coefficient is essentially affected by the paste characteristics. Similar findings were obtained by Bogas [2].

Hammer and Smeplass [47] performed rapid chloride penetrability tests (RCPT), according to ASTM C1202 [113], having concluded that the type of LWA was not significant for the chloride diffusivity of SLWAC. Through RCPT and immersion and salt ponding tests, Chia and Zhang [49] also found comparable pass charges and chloride penetration depths in SLWAC with expanded clay LWA and w/b of 0.35 and 0.55 and in NWC of the same composition. Through the examination of the split SLWAC specimens, less than 0.15% of free chloride ions were found in the interior of the LWA particles, suggesting that most chloride ions did not penetrate into the LWA. This was attributed to the dense outer shell of the LWA and to the high quality of the ITZ.

However, Real et al. [6] found that SLWAC with more porous LWA presented higher chloride migration coefficients than NWC of the same composition, especially for high w/b. Similar trends were obtained by Vieira [78] and Youm et al. [110]. Vieira [78] suggested that the previous vacuum saturation to which the specimens were subjected may have allowed the participation of the LWA in the chloride migration process. According to Bogas and Gomes [7], the weak influence of LWA should be more relevant for lower water content of concrete, given that, for just a small reduction in the RH of concrete (<5%), LWA is partly dried out (Section 2.3).

Based on accelerated RCPT performed in SLWAC with FA LWA and w/b of 0.32 and 0.35, Gesoğlu et al. [73,84] concluded that the increase replacement of NWA with LWA led to the increment of the passed charge. The same was witnessed by Hwang and Tran [109] in SLWAC with foamed LWA. The authors attributed the results to the high absorption capacity and open porosity of the LWA. On the other hand, taking into account RCPT, RCMT and salt ponding tests, Liu et al. [35] found that the chloride penetration resistance tends to decrease with the incorporation of coarse and fine LWA in SLWAC. In a study performed by Bogas and Gomes [89], SLWAC with volcanic scoria and w/c of 0.35 and 0.56 consistently presented higher chloride migration coefficients than NWC of similar composition. According to the authors, besides the high open structure of this type of LWA presents, as the RCMT were performed on sawn specimens, chlorides were able to penetrate through the sectioned LWA, near the surface.

Bogas and Gomes [7] carried out a comprehensive study in which the chloride penetration resistance of a wide range of SLWAC produced with different types, volumes and wetting conditions of expanded LWA, types of binders and w/c of 0.3–0.55, was analysed taking into account non-steady RCMT, immersion and salt-fog tests. Even taking into account different exposure conditions (submersion, drying and wetting) and chloride penetration mechanisms (migration and diffusion), the authors concluded that the chloride penetration resistance was little affected by the type of LWA. Actually, an exponential relation between the RCMT diffusion coefficient and the w/c was found, regardless of the type of LWA (Figure 8). However, SLWAC with very porous LWA showed diffusion coefficients up to 20% higher than NWC. The volume and wetting conditions of LWA showed little influence on the chloride resistance. Nevertheless, a slight reduction of 13% found on the chloride diffusion coefficient of SLWAC with initially dried LWA was attributed to the better quality attained in the ITZ. The authors also reported a greater long-term reduction of the diffusion coefficient in SLWAC with more porous LWA, which was attributed to the more effective internal curing and less self-desiccation. A good correlation was found between the different analysed tests.

Several authors suggest that the chloride penetration in SLWAC is usually lower than in NWC of the same strength level [35,82,114,115]. However, Al-Khaiat and Haque [64] found lower chloride penetration resistance in SLWAC with coarse and fine LWA than in NWC of equal strength. This is not corroborated by the study of Bogas and Gomes [7], which reported the same chloride migration coefficient, when natural sand was partly replaced by fine LWA in concrete with a w/c of 0.35. Similarly to carbonation resistance, the chloride penetration resistance is poorly related with the compressive strength of SLWAC [6,7]. In fact, the chloride penetration resistance is mainly dependent on the characteristics of the paste, while compressive strength may be highly influenced by the properties of LWA [6].

### 4.2. Influence of the Type of Binder

Various authors have reported that the incorporation of pozzolanic admixtures, such as SF and FA, improves the long term chloride penetration resistance of SLWAC, due to matrix porosity refinement [6,51,70,96,108,110,116,117,118]. However, Real et al. [6] found that, at 28 days, the incorporation of 15–30% FA in SLWAC with different types of LWA and w/b of 0.35–0.55 led to the increment of the chloride migration coefficient, due to the short wet-curing period and the early testing age. Nevertheless, after 1 year, the tendency was inverted, and SLWAC with FA performed better than SLWAC with OPC, due to further development of pozzolanic reactions [86]. The same tendencies were observed by Bogas [2], in SLWAC with 22–40% FA and a w/b of 0.35.

Youm et al. [110] reported a significant improvement of the chloride migration coefficient with the incorporation of 3.5–7% SF in SLWAC with a w/b of 0.25. The same was ascertained by Real et al. [6], in SLWAC with different types of LWA and w/b of 0.35–0.55 incorporating 6–9% SF, and by Wang et al. [119], in SLWAC with expanded slate, 10% SF and a w/b of 0.30. According to Real et al. [6], SF acts, not only in porosity refinement, but also on the composition of the pore solution, which affects the migration properties. The advantageous action of SF on the chloride penetration resistance was also confirmed by other authors [51,70], although there are still great divergences as to its level of contribution.

The influence of the incorporation of LF in SLWAC was assessed by Real [86], having observed higher chloride migration coefficients than in SLWAC with OPC. The author attributed the results to the reduction of hydration products compared to the same amount of OPC, given that this type of addition only contributes to the porosity refinement through the filler and nucleation effects.

Thomas [117] reported the results of RCPT and of non-steady-state diffusion tests on NWC and SLWAC with w/b of 0.3 and 0.40, 8% SF and 25, 40 or 56% FA. The results confirm the benefits of the addition of FA, with permeability and diffusion coefficients having been significantly reduced. After 3 years, the reduction of the apparent chloride diffusion coefficient reached as much as 70%. The author showed that the chloride penetration resistance of high performance concrete with low w/c and SF increases with the use of pre-soaked expanded slate LWA. This was attributed to the internal curing provided by the LWA, which may have compensated the self-desiccation of the concrete, especially at the ITZ. For this reason, higher chloride ion penetration resistance in high performance SLWAC than in the NWC counterpart is suggested.

### 4.3. Chloride Ingress in Marine Environment

Various investigations have been conducted in marine structures [1,16,118,120,121,122] and in specimens subjected to long-term real environmental exposure conditions [2,30,86,123,124,125]. In general, the chloride penetration resistance of SLWAC can be as high as that of NWC [30,64,118,120,121,125], which is in line with the laboratory studies presented in literature [2,6,35,49,50,51,70,86,108]. As previously discussed (Section 1), various examples of existing structures in Japan [106], Norway [16,79,126,127,128] and North American [1,120] show the high potential durability of SLWAC to chloride attack.

Polder et al. [121] reported lower chloride ingress in SLWAC than in NWC of similar compressive strength, subjected to 18 years of exposure. However, SLWAC was produced with higher binder content and lower w/b than NWC, which might justify the results.

Real and Bogas [125] performed a comprehensive experimental study, involving 65 slab specimens of SLWAC and NWC, covering a wide range of density (D1.6-2.0) and strength classes (LC20/22-LC55/60), subjected to different real exposure conditions, namely XS1 (exposed to airborne salt but not in direct contact with sea water zone), XS2 (permanently submerged zone) and XS3 (tidal, splash and spray zone). After 3 years of exposure, the authors found that the chloride diffusion of SLWAC generally tended to be similar to that of NWC of the same composition, at least for w/c up to 0.55. Thomas and Bremner [30] found equivalent chloride penetration behavior in SLWAC with expanded shale LWA and in NWC of the same age and composition, in concrete blocks with w/c of 0.4–0.6 and high amount of slag, exposed 25 years in the harsh tidal zone of a marine exposure site on Treat Island, in the coast of Eastport Maine. The same was mentioned by Moffatt et al. [123], for SLWAC with expanded shale and expanded clay LWA, FA and a w/b ratio of 0.38, after 21 years of exposure to harsh marine environment.

However, Real and Bogas [125] found that the chloride penetration was higher in SLWAC with more porous LWA or the absence of a dense outer shell, due to the greater surface chloride content usually observed in SLWAC than in NWC. In fact, depending on the environmental conditions and composition parameters, SLWAC presented a surface chloride content up to 2.9 times higher than NWC. The greater surface chloride content in SLWAC was also documented by other authors [14,30,35,129]. This may be due to a partial filling with sea water of the LWA pores near the concrete surface, increasing the surface chloride content [2,35,125,127]. In 90 day salt ponding tests performed in SLWAC with oil palm shell and a w/b ratio of 0.38, Teo et al. [62] also invoked the same reason to explain the higher surface chloride concentration found in SLWAC than in NWC. Nevertheless, Real and Bogas [125] reported comparable chloride penetration resistance in SLWAC with dense LWA and in NWC.

In addition, after 15 years in tidal zone, Osborne [129] witnessed slightly higher chloride ingress in SLWAC with FA or scoria LWA than in NWC of similar composition. Greater chloride penetration in SLWAC with fine and coarse LWA than in NWC of the same compressive strength was also found by Al-Khaiat and Haque [130], after 3 years of exposure to marine environment.

Contrary to gas transport, ion diffusivity is increased if concrete is saturated. Therefore, it is important to know the moisture distribution in SLWAC. LWAs are usually unsaturated due to self-desiccation or drying of SLWAC. Thus, the participation of the aggregates in the chloride ingress should be more effective for low-quality pastes and when saturation in submerged areas is high enough to establish continuous passages for water transport through the aggregates. In other words, if the aggregates are not initially saturated, the chloride ingress through the particles will only occur by permeability or, less effectively, by capillary absorption [2]. According to Kockal and Ozturan [51], if aggregates are pre-soaked, chloride ions may be quickly transported, due to their open porosity. This may explain why Real and Bogas [125] found lower chloride penetration in SLWAC with initially dry porous LWA than in SLWAC with pre-saturated denser LWA.

Real and Bogas [125] also investigated the influence of the w/b ratio and type and volume of mineral additions, such as SF, FA and LF. The authors confirmed that, in general, the chloride content increased with the w/b ratio, having attributed the results to the increment of concrete porosity and to the reduction of binding capacity, due to lower C_3_A hydration products and CSH content. In addition, the authors found unexpected high chloride penetration in SLWAC with SF, having justified the results with a lack of effective dispersion of this type of addition, which might not have had a significant contribution to the refinement of the concrete microstructure. However, for the incorporation of 6% SF, the chloride diffusion coefficient decreased about 11% and 60% in SLWAC and in NWC, respectively. This higher efficiency of SF in NWC than in SLWAC may be attributed to the easier dispersion of SF in NWC, the lower contribution of SF to the improvement the ITZ in SLWAC, which is already high quality, and the partial absorption of SF by LWA [6]. The higher chloride penetration in concrete with SF was attributed to a respective increase in surface chloride concentration. Higher efficiency of SF was reported by Gjørv et al. [131], in SLWAC with different types of LWA subjected to 3 years of natural seawater. The authors found that the incorporation of 9% SF reduced the chloride diffusivity by a factor of about 5, which was attributed to the better microstructure and to changes of the chemical properties of the pore solution. The authors also found that the type of fine aggregate and maximum aggregate size had a minor effect on the chloride diffusivity.

Real and Bogas [125] found the best performance in SLWAC with 30% FA, due to the development of pozzolanic reactions, which contributed to the refinement of the concrete microstructure [23,132] and to the increase of the binding capacity stemming from the higher amount of aluminates and CSH [133,134]. Compared to concrete with OPC, the chloride diffusion coefficient decreased about 50 and 70% in concrete with 15 and 30% of FA, respectively. Similar trends were reported by Alexander and Thomas [135] in concrete with 25% FA and with only OPC, after 25 years in XS3. Moffatt et al. [123] and Moffatt and Thomas [124] highlighted the benefits of the incorporation of high volumes of FA and SF to the chloride penetration resistance of SLWAC.

In a recent study, Moffatt and Thomas [123] investigated the long-term durability of modified density concretes, produced with different mineral admixtures, exposed for 25 years in the same tidal zone harsh environment considered in the study of Thomas and Bremner [30]. Concretes with both SF and FA and w/c of 0.4–0.6 showed a penetration of 0.05% of chlorides by mass of concrete at about 40 mm depth, while concretes without mineral admixtures presented up to 90 mm chloride penetration. The authors also reported that for SLWAC without mineral admixtures, reducing w/b from 0.6 to 0.5 resulted in a 3.5 times lower diffusion coefficient, while the presence of mineral admixtures reduced the diffusion coefficient by 7 times.

As expected, Real and Bogas [125] found that the incorporation of 15–30% LF in SLWAC led to higher chloride penetration than in SLWAC with OPC, given that the contribution of this type of mineral admixture to microstructural refinement through the filler and nucleation effects was not offset by the reduction of cement hydration products and of its non-chloride binding capacity.

Real and Bogas [125] reported that after 3 years of exposure to classes XS2 and XS3, the chloride content at the 30 mm reinforcement depth was about 0.80 and 1.57 wt% of binder, respectively. Although the critical chloride content can vary in a wide range, at these values, corrosion was likely to have started, at least, in slabs subjected to XS3. However, visual observation of the SLWAC and NWC slabs revealed no signs of corrosion. After 15 years of exposure to a tidal zone, no evidence of corrosion was also reported by Osborne [129] in SLWAC with FA.

The chloride penetration is still a source of some uncertainty for both SLWAC and NWC, namely concerning natural environment exposure, as demonstrated by the contradictory results reported in literature.

## 5. Service Life Prediction for Carbonation and Chloride-Induced Corrosion

Service life is defined in current standards using either a prescriptive methodology or a performance based methodology. In terms of prescriptive methodology, in European countries, EN 206 [22] recommends composition limits for maximum w/c, minimum cement content and minimum compressive strength. This is complemented by EN 1992-1-1 [18], which defines the minimum concrete cover for reinforcement to ensure, at least, 50 years of service life. However, EN 206 [22] only accounts for NWC and no recommendation is provided for SLWAC. In turn, EN 1992-1-1 [18] determined that, for SLWAC, 5 mm should be added to the minimum concrete cover defined for NWC, regardless of the exposure class (Table 3).

In Portugal, LNEC specification E 464 [136] is an extension of the EN 206 requisites. This specification provides recommendations for both NWC and SLWAC produced with different types of binder (Table 3). However, the suggested limits are still poorly substantiated. For instance, as discussed, SLWAC durability should not be determined as a function of mechanical strength (Section 3.1 and Section 4.1).

On the other hand, performance-based methodologies allow service life prediction based on concrete performance. However, service life is governed by the deterioration mechanisms, which are influenced by several parameters, such as concrete composition and curing, exposure and testing conditions. The complexity of these mechanisms explains why simplified models have been suggested for service life design.

**Figure 9:**
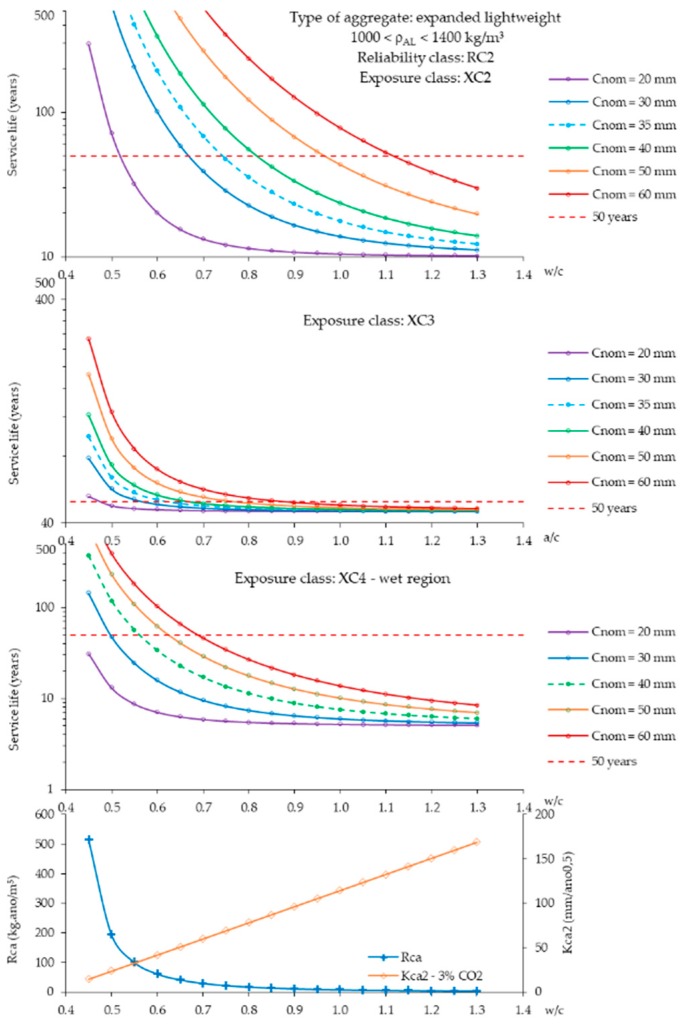
Example of abacuses for the service life prediction of concrete under carbonation-induced corrosion (adapted from [86]).

Ferrer et al. [101] investigated the service life of SLWAC under carbonation-induced corrosion. The authors resorted to the semi-probabilistic performance-based approach suggested by LNEC specification E 465 [137] to predict the service life of SLWAC. Based on the partial safety factor approach of E 465 [137], new abacuses were defined for the prediction of the service life of uncracked concrete as a function of the concrete cover and the w/c or accelerated carbonation coefficient (Figure 9). These abacuses were determined for each carbonation exposure class and for a given reliability class associated to a maximum probability of failure of 6.7%. The adopted procedure is described in detail in [101].

Moreover, limiting values of SLWAC composition for different carbonation exposure classes were recommended for an intended service life of at least 50 years under carbonation-induced corrosion (Table 4). The limits indicated in Table 4 do not take into account the beneficial effect of stage 1 of the biphasic carbonation behaviour of SLWAC (Section 3.2).

The abacuses and prescriptive compositions were defined according to different density classes of the most common structural LWA (expanded and sintered FA). The abacuses are valid for concretes produced with blended cements, namely with FA, SF and LF, provided that only the w/c is considered, without including the contribution of the mineral admixtures.

The abacuses show that common SLWAC can present significantly lower service life than NWC or SLWAC with dense LWA. In fact, the maximum w/c of SLWAC with porous LWA (density <1000 kg/m^3^) is up to 0.2 higher than that of NWC, while for dense LWA (density >1400 kg/m^3^), the recommended composition is identical.

However, the authors concluded that, even for SLWAC with matrixes of low to moderate quality (depending on the type of LWA, w/c up to 0.5–0.6, for the most aggressive class XC4), carbonation-induced corrosion should not be the dominant degradation mechanism, provided that appropriate concrete cover thicknesses are considered. This is in line with the work of Schulze and Günzler [57], which estimated that the average carbonation of SLWAC with w/c below 0.65 would take more than 50 years to reach 30 mm under air-dry conditions. Similar findings were reported by Bogas and Gomes [89], even taking into account SLWAC with very porous volcanic LWA.

Table 5 presents the additional concrete cover in SLWAC (Δc_min_) relative to the minimum concrete cover defined in EN 1992-1-1 [18], for an intended service life of 50 years under carbonation-induced corrosion. Only SLWAC with LWA of density >1400 kg/m^3^, require less than additional 5 mm of concrete cover, as recommended in EN 1992-1-1 [18].

**Figure 10:**
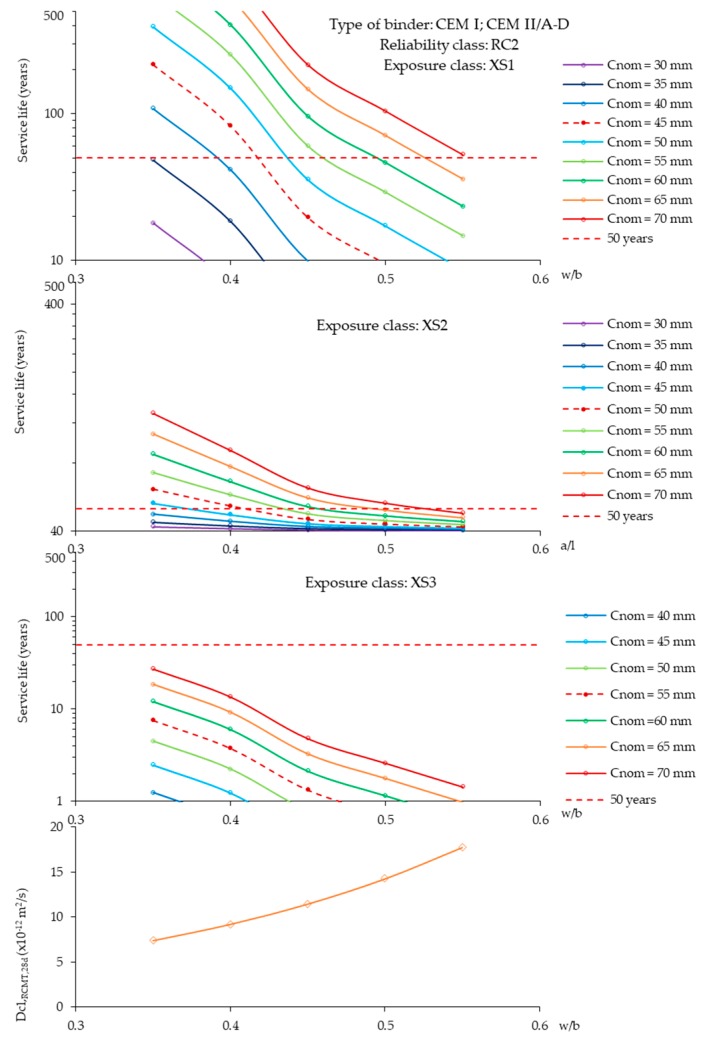
Example of abacuses for the service life prediction of concrete under chloride-induced corrosion (adapted from [86]).

Real et al. [138] studied the chloride-induced corrosion service life of SLWAC produced with different types of LWA and types and contents of binder. The authors compared the semi-probabilistic performance-based approaches suggested by LNEC specification E 465 [137] and Duracrete R15 [139], having found that, except for class XS1, Duracrete R15 [139] tends to provide more conservative estimations than E 465 [137]. Furthermore, the authors resorted to E 465 [137] to propose abacuses that can roughly predict the service life of uncracked SLWAC as a function of the concrete cover and the w/b or chloride migration coefficient (Figure 10). These abacuses were defined for each chloride exposure class, taking into account a reliability class that corresponds to a maximum probability of failure of 6.7%. The adopted procedure is described in detail in [138].

Based on the findings of previous studies (Section 4.1), the chloride migration coefficient adopted in the service life prediction model was defined by a unique relation, as a function of the w/c, regardless of the type of expanded LWA. For SLWAC with sintered FA, a new relation was defined. In addition, in order to take into account different binders, the authors adopted the concept of equivalent w/c. This parameter basically represents the w/c of blended cement with the same diffusion coefficient of concrete produced with only CEM I [138]. Then, the service life of blended cements can be estimated from the same abacus defined for OPC mixtures, using the converted equivalent w/c.

Limiting values of concrete composition were also proposed for the most common uncracked SLWAC, for an intended service life of at least 50 years under chloride-induced corrosion (Table 6). As expected, given that the chloride migration coefficient of diffusion is not affected by the type of aggregate, SLWAC does not require lower w/c or additional concrete cover when compared to NWC. Only for SLWAC with FA LWA w/c is up to 0.05 lower. However, according to Real [86], based on the results obtained in [107], a non-conservative simplification is introduced in the service life models of E 465 [137] and Duracrete R17 [140], because these documents do not account for the influence of the type of aggregate in the surface chloride concentration. In this case, a lower service life would be expected for SLWAC compared to NWC.

In sum, the above mentioned studies concerning the carbonation and chloride induced corrosion service life prediction, allowed the definition of guidelines for the production of durable SLWAC, improving the prescriptive philosophy adopted in current standards.

## 6. Influence of Cracking on SLWAC Durability

Various investigations have been carried out regarding the influence of cracking on the durability of NWC [141,142,143,144], involving experimental or numerical analysis. However, so far, knowledge on the durability behaviour of cracked SLWAC is very limited.

Bremner et al. [54] analysed the effect of stress on the nitrogen gas permeability of SLWAC and NWC with w/c of approximately 0.6. The authors verified that the flow rates tended to remain constant up to a critical stress corresponding to the onset of unstable crack propagation, from which the flow rate rapidly increased. A rapid increase in permeability occurred at lower levels of applied stress-to-strength ratio in NWC (54 to 62%) than in SLWAC (72 to 82%). Sugiyama et al. [68] reached similar conclusions testing concretes with w/c of 0.4 and 0.6.

The EuroLightConR13 [145] presents a large-scale chloride penetration test programme performed on concrete beams made with expanded clay LWA and sintered FA LWA, as well as with NWC. The beams were exposed to alternating moisture and temperature cycles and have been tested under unrestrained and restrained conditions, in order to make them susceptible to temperature-induced microcracking. After 6 months of testing, the results revealed that SLWAC performed as well as NWC, under both restrained and unrestrained conditions.

Noteworthy is the study conducted by Bogas et al. [146], which assessed the influence of cracking on the capillary absorption and the accelerated carbonation resistance of SLWAC produced with distinct types of aggregate and w/c. The authors analysed the effect of artificial and natural cracks with 0.1–0.3 mm on these properties of SLWAC, as well as its relative behaviour against cracked NWC of equal composition. In order to compare absorption coefficients of concrete with different crack lengths, the authors proposed a new crack parameter, which can be applied to any type of NWC and SLWAC. The influence of cracking in absorption was found to be less significant when the average water height is at least two times higher than the crack length. The authors concluded that the type of aggregate did not significantly affect the influence of artificial cracks on the studied durability properties, regardless of the crack width. Nevertheless, the influence of cracking on absorption and carbonation slightly decreased with the increasing porosity of the LWA, which suggests that the durability properties in NWC are more affected by the artificial cracking than in SLWAC. However, the influence of natural cracks was higher in SLWAC with more porous LWA. According to the authors, when LWAs are intercepted by natural cracks, the gas permeability is increased and LWA can better participate in the carbonation mechanism. For this reason, contrary to NWC, the influence of natural cracks in the carbonation rate of LWAC was not higher than that of artificial cracks.

Based on the obtained results, depending on the ratio carbonation depth/crack length, the carbonation rate under real exposure conditions could increase more than 80% in cracked concrete [146]. Nevertheless, according to ACI 213R [56], the concrete cover in SLWAC is expected to maintain its low-permeable integrity, despite the accumulation of shrinkage, thermal and structural load related.

## 7. Conclusions

Despite the various laboratory and field investigations which have been carried out, our perfect knowledge on the durability behaviour of SLWAC is still a long way off. The paste composition, type of aggregate, curing and exposure conditions, test setup, penetration mechanism and concrete water content are some factors that help explain the different tendencies reported in the literature.

Due to the higher porosity of the LWA, the carbonation resistance of SLWAC tends to be lower than that of NWC of the same composition. However, considering the biphasic carbonation model and the protection provided by the paste, the carbonation resistance of less porous SLWAC can be comparable to that of NWC in real environment.

Regarding concrete under chloride attack, although the knowledge is still limited, investigation works either performed in the laboratory or in real environments indicate that SLWAC can have similar to better durability performance than NWC, especially when the same strength level is considered. However, although SLWAC and NWC of identical composition present similar chloride diffusion coefficients, the surface chloride content of SLWAC tends to be higher than that of NWC, which may lead to higher long-term chloride penetration.

The importance of the quality of the paste over the characteristics of the LWA in durability performance was highlighted. An effective dispersion and involvement of the LWA particles by high quality paste is necessary to ensure the good durability performance of SLWAC.

Durability standardization regarding SLWAC is still insufficient, being one of the main gaps of present knowledge in this domain. The calibration of the model parameters used in service life prediction models is necessary, adapting them to SLWAC, which requires comprehensive studies involving laboratory and long-term real-environment tests. Therefore, further research is needed in this domain, contributing to a better prediction of the durability behaviour of SLWAC and a greater confidence in using this type of concrete.

## Figures and Tables

**Table 1:** Carbonation coefficients of stage 1 and 2, K_ca1_ and K_ca2_, of the biphasic carbonation model, for SLWAC with different types of LWA as a function of the w/c (adapted from [101]).

Type of Aggregate	Aggregate Density Class	K_ca1_ (mm/year^0.5^) ^a,b^	K_ca2_ (mm/year^0.5^) ^b^	w/c Validity Range
Expanded LWA	<1000 kg/m^3^	136.7w/c-54.4 (for w/c > 0.45, disregard K_ca1_)	220.8w/c-81.1	0.45–0.93
	>1000 kg/m^3^ and <1400 kg/m^3^		181.9w/c-67.4	0.45–1.30
	>1400 kg/m^3^		128.0w/c-43.1	0.45–1.30
Sintered FA LWA	>1300 kg/m^3^		159.2w/c-50.3	0.45–0.93

^a^ Coincides with K_ca_ of NWC; ^b^ Valid for concrete with CEM I, as well as CEM II with SF, FA and FL.

**Table 2:** Carbonation depths of SLWAC exposed to natural conditions.

Reference	Exposure Conditions	Description		SLWAC Characteristics	Exposure Period (years)	Carbonation Depth (mm)
Holm et al. [102]	XC4	Main deck	of concrete ships built during World War II in Chesapeake Bay at Cape Charles, Virginia		>50	1
	XC4	Exposed wing walls				1–2
	XC4	Hull and bulkheads				0
Holm and Bremner [1]	XC4	Top	of the bridge deck of Chesapeake Bay Bridge		35	2–8
	XC3	Underside				2–13
	XC4	Deck surface of the Interchange Bridge at Coxsackie			15	5–10
Ohuchi et al. [106]	XC4	Japanese bridges and viaducts		Compressive strengths of 23–26 MPa	19	13–16
Bandyopadhyay and Swamy [104]		SLWAC with expanded shale LWA, exposed to an industrial environment		w/c of 0.53	2	1
Mays and Barnes [14]		6 concrete structures in the United Kingdom			20	6–25
Short and Kinniburgh [58]					3	2.5
Bogas [2]	XC4	in port facilities in Setúbal, Portugal		w/b of 0.35 and 0.45	2	0–2
Real [86]	XC3	in Grilo tunel in Lisbon, Portugal		w/b of 0.55 and 0.65 types of binder: 6–9% SF, 15–30% FA and 15–30% LF	3	8–27
	XC3	on top DECivil building, in Instituto Superior Técnico, in Lisbon, Portugal				6–28
	XC4					4–21
	XC4/XS1	Cascais marina, Portugal			2.5	5–28

**Table 3:** Composition limits prescribed by various normative documents for a service life of 50 years.

Normative Document	Parameter	Exposure Conditions						
		Carbonation				Chlorides from Sea Water		
		XC1	XC2	XC3	XC4	XS1	XS2	XS3
EN 206 [22]	Maximum w/c	0.65	0.6	0.55	0.5	0.5	0.45	0.45
	Min. strength class	C20/25	C25/30	C30/37	C30/37	C30/37	C35/45	C35/45
	Minimum cement content (kg/m^3^)	260	280	280	300	300	320	340
EN 1992-1-1 [18]	Minimum cover (mm)	15 ^b^	25 ^b^	25 ^b^	30 ^b^	35 ^b^	40 ^b^	45 ^b^
E 464 [136]	Maximum w/c	0.65	0.65	0.55–0.60 ^a^	0.55–0.60 ^a^	0.45–0.55 ^a^	0.45–0.55 ^a^	0.40–0.45 ^a^
	Minimum strength class	C25/30 LC25/28	C25/30 LC25/28	C30/37 LC30/33	C30/37 LC30/33	C30/37-C40/50 ^a^ LC30/33-LC40/44 ^a^	C30/37-C40/50 ^a^ LC30/33-LC40/44 ^a^	C35/45-C50/60 ^a^ LC35/38-LC50/55 ^a^
	Minimum cement content (kg/m^3^)	240–260 ^a^	240–260 ^a^	280–300 ^a^	280–300 ^a^	320–360 ^a^	320–360 ^a^	340–380 ^a^

^a^ Depending on the type of binder; ^b^ Values defined for NWC, for the S4 structural class (5 mm should be added in SLWAC).

**Table 4:** Minimum w/c for SLWAC with intended design service life of 50 years under carbonation-induced corrosion, proposed by [101] *.

Type of Aggregate	Density of LWA (kg/m^3^)	Exposure Class			
		XC1 (c_nom_ = 25 mm)	XC2 (c_nom_ = 35 mm)	XC3 (c_nom_ = 35 mm)	XC4 (c_nom_ = 40 mm)
Expanded LWA	<1000	w/c > 0.65	w/c > 0.65	w/c > 0.55	w/c > 0.50
	>1000 and <1400	w/c > 0.70	w/c > 0.70	w/c > 0.60	w/c > 0.55
	>1400	w/c > 0.85	w/c > 0.85	w/c > 0.65	w/c > 0.60
Sintered FA LWA	>1300	w/c > 0.70	w/c > 0.70	w/c > 0.60	w/c > 0.50

* Valid for CEM I, CEM II/A, CEM II/B, CEM IV/A and CEM IV/B.

**Table 5:** Additional concrete cover in SLWAC (Δc_min_) relative to the minimum concrete cover defined in EN 1992-1-1 [18], for intended design service life of 50 years under carbonation-induced corrosion*.

Type of Aggregate	Density of LWA (kg/m^3^)	Exposure Class		
		XC2	XC3	XC4-RH
		Δc_min_ (mm)		
Expanded LWA	<1000	18	16	24
	>1000 and <1400	11	9	14
	>1400	4	2	5
Sintered FA LWA	>1300	9	9	16

* Valid for CEM I, CEM II/A, CEM II/B, CEM IV/A and CEM IV/B.

**Table 6:** Minimum w/c for SLWAC and NWC with intended design service life of 50 years under chloride-induced corrosion, proposed by [138] *.

Type of Aggregate	Exposure Class		
	XS1 (c_nom_ = 45 mm)	XS2 (c_nom_ = 50 mm)	XS3 (c_nom_ = 55 mm)
Expanded LWA and NWA	w/c > 0.45	w/c > 0.40	w/c > 0.30
Sintered FA LWA	w/c > 0.40	w/c > 0.35	w/c > 0.30

* Valid for concrete with CEM I.
